# Study protocol for a randomised evaluation of a brief psychological intervention for clinically stressed parents of adolescents: Approach for Parenting Teenagers (APT)

**DOI:** 10.1186/s40359-026-04048-w

**Published:** 2026-03-21

**Authors:** Alex Desatnik, Catherine Jones, Peter Fonagy, Julia Britton, Victoria Hayward, Ruth Glover, Lara Taylor, Nicolas Lorenzini

**Affiliations:** 1 University College London, London, UK; 2 Open Door Young People’s Consultation Service, London, England, UK

**Keywords:** Parenting, Intervention, Stress, Adolescence, Randomised controlled trial

## Abstract

**Background:**

Adolescence can be a stressful and difficult period for young people and parents, with many changes occurring during this time, and a heightened risk of mental health problems for adolescents. The quality of parenting an adolescent receives and the relationship between parents and adolescents is of particular importance during this time and predicts adolescent outcomes. Parenting is a modifiable risk factor which can be targeted to improve outcomes for adolescents and their families. Many existing parenting interventions are group-based, which is not appropriate for all parents, leading to disengagement and dropout. Open Door created an intervention, the Open Door Approach to Parenting Teenagers (APT), an individual parenting programme for clinically stressed parents of adolescents. APT comprises six weekly 50-minute appointments, working with individuals or couples. To increase flexibility and maximise engagement, this intervention will be delivered both face-to-face and online. The preliminary evaluation of APT yielded promising findings for the feasibility, acceptability, and real-world effectiveness of APT. This randomised evaluation is a continuation of this.

**Methods:**

Participants will be parents of adolescents who self-refer to Open Door, reporting clinically significant levels of parenting stress through the SIPA. Participants will be randomly allocated to receive the APT intervention or waitlist control. The primary outcome measure will be the SIPA, measuring parenting stress, the parent-adolescent relationship, adolescent and parent wellbeing, and mental health outcomes. Secondary outcome measures measure mediators of change and adherence to APT. Results on outcome measures will be compared between the APT group and control group immediately following the intervention, three months post intervention, and six months post intervention.

**Discussion:**

If APT is an effective intervention compared with the waitlist control, this could improve outcomes for adolescents and parents, and be an accessible and engaging option for parents of adolescents.

**Trial registration:**

This trial is registered at the International Council on Harmonisation of Technical Requirements for Registration of Pharmaceuticals for Human Use Good Clinical Practice (ICH GCP), (Clinical Trial NCT03916172).

**Supplementary Information:**

The online version contains supplementary material available at 10.1186/s40359-026-04048-w.

## Introduction

### Background and rationale

Adolescence can be a challenging time for young people and their families, with many changes and difficulties often arising during this period. Adolescents have the highest rates of mental health difficulties of any age group, yet many adolescents do not access appropriate support [1]. During adolescence, conflict between adolescents and parents has been found to increase, affecting well-being and self-esteem [2, 3]. The challenges of parenting an adolescent can bring an increase in parental stress [4], which can impact parents’ health [5]. One effective way to improve outcomes for adolescents is to target and improve the quality of parenting an adolescent receives and improve the parent-adolescent relationship. Parenting interventions can reduce challenging and risk behaviours in adolescents, improve the parent-adolescent relationship, and reduce parenting stress [6, 7]. However, to date, the majority of parenting interventions are aimed at parents of younger children and are delivered in a group format. Group interventions can bring challenges, with parents reporting feeling judged and pressured to participate, and describing the stigma of attending a group parenting intervention a barrier to engagement [8, 9]. There is a need for an individual parenting intervention that can facilitate an improved therapeutic alliance, aiding treatment engagement [10]. Engagement difficulties have also been found in face-to-face interventions due to the time constraints attached to attending in person and the perceived stigma of being seen at a parenting intervention [11]. Digital interventions allow for increased privacy and time flexibility, as parents are not required to travel to and from sessions, with the potential to increase engagement [11, 12]. As parents’ preferences for face-to-face or digital interventions have been mixed, there is a need for a parenting intervention offering a flexible approach of both face-to-face and online sessions to meet preferences and engage more individuals [13].

This paper outlines the protocol for a randomised controlled trial (RCT) to measure the effectiveness of Open Door’s APT intervention. APT is a brief, manualised parenting intervention for clinically stressed parents of adolescents, with a particular focus on the adolescent-parent relationship. APT can be delivered face-to-face or digitally over six sessions. APT follows the Open Door APT manual, which is a six-session model. The APT manual contains guidance on applying APT to practice, core techniques, and step-by-step guides for sessions one to six as well as working with the adolescent and parent, working with couples, and additional considerations. Clinicians use the manual and follow a checklist for each session, which contains actions for each session.

Findings from its preliminary evaluation, a pre-post pilot study of 279 parents, demonstrated promising results, particularly in terms of feasibility, acceptability, and real-world effectiveness [14]. However, a significant limitation of this study was the lack of a control group and follow-up measures to demonstrate long-term effectiveness. Furthermore, as highlighted in intervention development frameworks [15, 16], an RCT is essential to establish the efficacy of an intervention and elucidate the mechanisms by which it achieves its effects. This trial will randomly assign parents to either an active group (complete the APT intervention immediately) or control group (placed on a waitlist). A waitlist control comparator group was chosen to ethically allow all participants access to the APT intervention while providing a baseline comparison against immediate treatment. Results on outcome measures will be compared between groups after the intervention, and at three and six month follow up intervals. This trial therefore builds on the pre-post study through ruling out spontaneous recovery and capturing long-term improvements. This trial will provide robust evidence regarding the effectiveness of a brief, individualised parenting intervention tailored for parents of adolescents. If effective, APT has the potential to offer a scalable solution to support parents and improve outcomes for adolescents and their families. By presenting this RCT protocol, this paper lays the groundwork for the next crucial step in establishing APT as a robust, evidence-based intervention.

### Objectives

The primary objective of this study is to evaluate whether APT is more effective than a waitlist control in reducing parental stress among clinically stressed parents of adolescents. Secondary objectives include assessing the impact of APT on parent-adolescent relationships, adolescent mental health, parental mental health, and parenting practices. Additionally, the study aims to evaluate the maintenance of these effects at three-month and six-month follow-up intervals. Mediators and moderators of treatment effects will also be explored to better understand the mechanisms of change. All participants will receive the APT intervention either immediately or after allocation to the waiting list, which may directly benefit the service user in reducing parental stress and improving the parent-adolescent relationship. Participants randomised into the APT intervention will benefit from receiving treatment earlier. We aim to assess engagement and acceptability, treatment fidelity and adherence to the APT model, and disseminate study outcomes to inform policymakers, commissioners and providers, to offer an effective evidence-based option for parents of adolescents.

## Methods: patient and public involvement, trial design

### Patient and public involvement

In planning this study we have been primarily guided by pressures the service faced from the local service user groups, the council and the CCG, to broaden the treatment options available for parents, with a need for individual face to face interventions. The APT intervention was developed through an ongoing consultation and collaboration with these bodies, who helped to identify the needs of the parents, allowing us to develop appropriate toolkits to include in the intervention. Through all stages of development of the intervention and the manual, ongoing feedback was sought from service users. This included the contents of the intervention, its length, and its usefulness, leading to the development of an intervention that reflected service users’ needs. For the preparation of the study design we consulted with both adolescent and parent service users to determine and assure relevance of the study, ways of involving both the parents and the adolescents in the research, and identify the most appropriate means of data collection. As a result of this consultation, the measures routinely used by Open Door were made available on both pen and paper and online/mobile, and an appropriate compensation model was developed. A steering group consisting of service users, members of the public, clinicians, academics and Open Door’s executives was created. Drawing upon the diversity of expertise, the members were instrumental in assisting us with the design of the study.

### Trial design

We propose a multi-site, 2-arm phase one superiority RCT, comparing APT to waitlist control, with an allocation ratio of 1:1.

## Methods: participants, interventions, and outcomes

### Trial setting

Recruitment for and delivery of the trial will be carried out in the United Kingdom at two clinical sites of Open Door Young People’s Consultation Service in a North London Borough.

### Eligibility criteria

Primary participants will be parents of adolescents who self-refer to Open Door. Adolescents will also be invited to take part in the study.

To ensure a focused and appropriate participant group, specific inclusion and exclusion criteria will be applied. Inclusion criteria requires that participants are parents of adolescents aged 11–18 years, with the adolescent residing with the parent for a minimum of two days per week. Additionally, parents must meet clinically significant levels of stress, as measured by the SIPA, and be proficient in English, to facilitate engagement with the intervention and assessment processes.

Exclusion criteria are designed to account for factors that could compromise the reliability of the study or interfere with the intervention’s applicability. These criteria exclude parents currently receiving treatment for psychotic illness or those who have previously received the APT intervention. Parents of adolescents with severe developmental disorders or life-threatening health conditions are excluded, as are those with adolescents currently receiving individual treatment at Open Door, to avoid potential overlap or confounding effects from simultaneous interventions.

The therapists providing APT have qualifications in psychology, psychotherapy or family therapy, and will undertake training and supervision in the APT model.

### Intervention and comparator

The aim of APT is to help the parent in the parenting of their adolescent and improve the adolescent-parent relationship. APT facilitates a collaborative relationship between the therapist and parent to develop parenting strategies, provide psychoeducation, and practice and reflect on behavioural experiments. Through this, APT aims to create a more balanced relationship between the parent and adolescent, where the adolescents’ views and feelings are considered, communication, boundaries and the relationship between parent and adolescent is explored, and the parent develops an enhanced understanding of their parental identity and role.

The APT intervention can be delivered one-to-one or with a couple. Participants will receive six weekly 50-min appointments, with the option of a 7th review session. Adolescents will be invited to attend one session; this is optional, and adolescents are not required to attend. The sessions will be delivered in person at one of the two clinical sites, or via video appointment. This allows for meeting parents’ delivery preferences to increase accessibility to the intervention.

Participants will be randomised into the APT intervention group or waiting list control group. Those randomised to waitlist will receive the APT treatment after no more than 25 weeks. Following randomisation, participants in the APT group will begin treatment. After six sessions of APT, the end-of-treatment battery will be administered to all participants (those in both APT and WL groups). The same battery will be administered to all participants again three months and six months following the end of treatment. After three months of randomisation, participants in the WL condition will be offered APT. These parents will be administered all test batteries from pre-treatment to six-month follow-up. Participants can withdraw from the study at any time. No concomitant care is permitted during the trial.

Fidelity of APT delivery to the APT model will be assessed through checklists completed by observers (see Outcomes section).
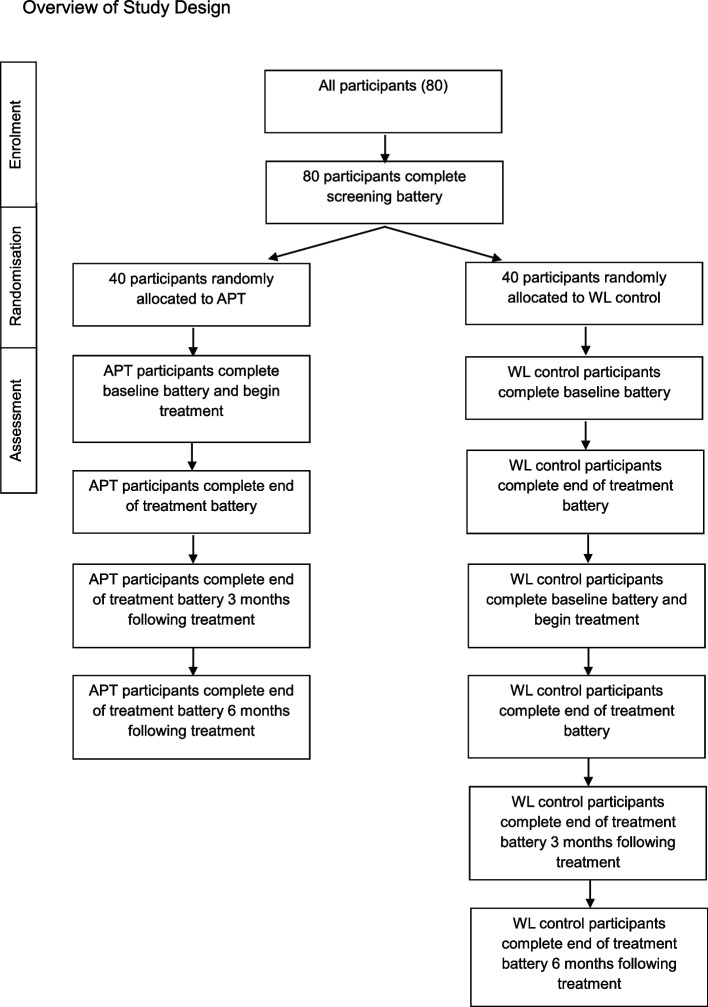


### Outcomes

The primary outcomes of interest in this study are changes in parental stress, the parent-adolescent relationship, and adolescent and parent wellbeing and mental health outcomes. These outcomes are measured by the primary measure, the SIPA, included in the parent battery. This is a 112-item self-report questionnaire structured into three main domain scores: Adolescent Domain; Parent Domain and Adolescent Parent Relationship Domain. A composite score (The Index of Total Parenting Stress) is computed from scores from all subsections. Scores are then classified into broad ranges: normal, borderline, clinically significant and clinically severe [17, 18].

Secondary outcome measures have been selected to explore mediators of change and adherence to the APT treatment model. The measures are grouped into four batteries: Parent, Adolescent, Psychotherapist, and Observer. Trial outcomes have been chosen due to their good psychometric properties and wide use in clinical practice and research [17–31].

### Parent battery

Strengths and Difficulties Questionnaire (SDQ): A brief behavioural questionnaire about the parent’s child. This contains 25 items, each scored from 0–2, measuring emotional symptoms, conduct problems, hyperactivity/inattention, peer relationship problems and prosocial behaviour. The total difficulties score is the sum of the first four subscales, with cut-offs as 0–13 within normal range, 14–16 borderline, 17–40 abnormal. The prosocial scale is scored separately, with higher scores indicating increased social behaviour [19].

Patient Health Questionnaire, 9-item depression module (PHQ-9): Each item is scored between 0–3, the total score is between 0–27, with 0–9 indicating mild low mood, 10–14 moderate low mood, 15–19 moderately severe low mood, 20–27 severe low mood [20, 21].

Generalised Anxiety Disorder Screener (GAD-7): Each of the 7 items is scored between 0–3, the total score is between 0–21, with 0–9 indicating mild anxiety, 10–14 moderate anxiety, 15–21 severe anxiety [22, 23].

Alabama Parenting Questionnaire-Short Form (APQ-SF): A 12-item, 5-point Likert self-report measure depicting parenting behaviours. This measures five dimensions of parenting: positive involvement with children, supervision and monitoring, use of positive discipline techniques, consistency in using positive discipline and use of corporal punishment. Higher scores on positive subscales indicate positive parenting behaviours, higher scores on negative subscales indicate poorer parenting behaviours [24–26].

Conflict Behaviour Questionnaire (CBQ): A 20-item questionnaire assessing perceptions of communication and conflict in the parent-adolescent relationship. Scores range between 0–20, with 1 point for responses indicating poor communication and conflict. Higher scores indicate higher levels of poor communication and conflict. We will be using the parent-report and adolescent self-report versions [27].

Parenting Scale: A 30-item questionnaire with a 7-point Likert scale measuring two parental discipline styles: Laxness and Over-reactivity, and measuring verbosity. Higher scores indicate poorer discipline practices [28, 29].

Reflective Function Questionnaire (RFQ): A brief screening measure of reflective functioning. This contains 8 self-report items with a 7-point Likert scale measuring certainty and uncertainty about mental states [30].

Working Alliance Inventory-Short Form-Client (WAI-SF-C): This measure will be used in the end-of-treatment battery. This is a 12 item, 7-point Likert scale, capturing three aspects of the therapeutic alliance: (a) agreement on therapy tasks, (b) agreement on therapy goals (c) development of an affective bond. Higher scores indicate a stronger therapeutic alliance [31, 32].

Goal Based Measure: Developed by CORC [33], used to rate patients’ accomplishment of their goals between zero to ten throughout treatment. Progress is tracked with zero indicating no progress, and ten indicating the goal has been achieved.

### Adolescent battery

Strengths and Difficulties Questionnaire Young Person Version (SDQ-YP): The self-report version of the SDQ. This also has 25 items, with each scored between 0–2, and measures emotional symptoms, conduct problems, hyperactivity/inattention, peer relationship problems and prosocial behaviour. Two versions that will be used: the 11–17 and the 18 +, depending on the age of the adolescent. The interpretation of the scoring is the same as the SDQ parent version.

Alabama Parenting Questionnaire Young Person Version (APQ-YP): This measures the adolescents’ perceptions of the parenting they receive. This also has 42 items, but ten are repeated to capture the adolescent's perception of both parents separately, with each item having a 5-point Likert scale. The interpretation of the scoring is the same as the APQ parent version.

Reflective Function Questionnaire (RFQ) [30] – See above.

### Psychotherapist battery

Working Alliance Inventory-Short-Form-Therapist (WAI-SF-T): a 12-item, seven point-Likert scale measuring the alliance elements described in the Parent Battery version. The interpretation of scoring is the same as the WAI-SF-C. This will be completed by APT therapists at the end of the treatment.

### Observer battery

To be administered by trial coordinators on a randomly selected intervention session's video:

Adherence to the APT model: Checklists to be filled in by observers for each of the six sessions, scoring one session per participant. Scoring will be performed by supervisors on a randomly selected intervention session video. The checklists capture actions for the therapist to undertake and components capturing the therapeutic stance, including level of activity of the therapist. Ten of these sessions will be rated by both coordinators to obtain appropriate levels of inter-rater reliability (> 0.9).

### Harms

During the consent process, it is explained to participants that any information that they disclose is confidential, with some exceptions, including if they disclose risk of imminent harm to themselves or someone else. For any service users presenting with high risk or safeguarding issues, Open Door’s standard procedure will be followed alongside the network to approach in case of emergencies, urgent referrals and safeguarding concerns. All research participants will be working with a trained mental health professional, who will receive weekly supervision with a highly specialised mental health professional. Should any unexpected risks or burdens arise, the participant will be able to discuss this with their therapist, who can discuss it with their supervisor and the research team (if necessary) to provide an appropriate solution.

### Participant timeline

(Fig. 1).

**Fig. 1:**
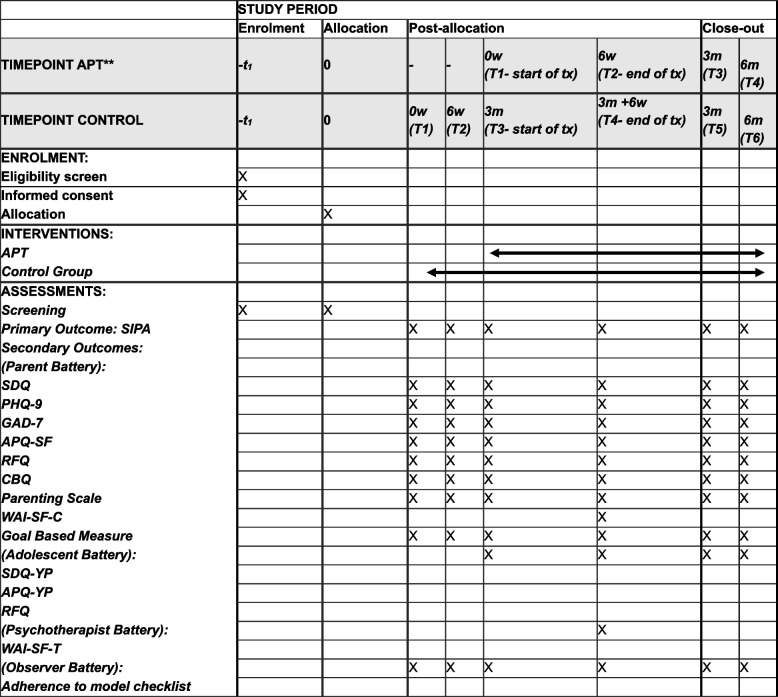
Schedule of enrolment, interventions, and assessments*

#### Sample size

Sample sizes are calculated based on the primary outcome measure (SIPA) and our goal of ascertaining superiority of APT. The target sample size for this study is 80 participants, divided equally between the APT intervention and waitlist control group (40 participants per arm). This sample size provides 80% power to detect a medium-to-large effect size (d = 0.4) at a significance level of α = 0.05, while accounting for an anticipated attrition rate of 32.5%, as informed by the pilot study findings [14]. This sample size ensures that the study is adequately powered to evaluate the primary outcome of parental stress reduction. This sample size is achievable and able to capture the difference between arms.

#### Recruitment

The pathway for parents participating in the RCT is similar to the current parent support pathway at Open Door, with parents being referred from other NHS services or self-referring. Upon referral, parents will be placed onto a waitlist, and prior to inclusion in the trial they will be contacted by a Research Assistant (RA) to ascertain suitability to participate. Participants will receive a phone call to inform them of the study and offer the opportunity to participate. Once consent to participate is obtained parents will complete the baseline battery and be given a participant number to identify participants anonymously. Participants randomised into APT will start treatment immediately, providing a further incentive to agree to randomisations. Those randomised to waitlist will receive the APT treatment after no more than 25 weeks. Once randomised, parents will be asked for consent to contact their adolescent and if granted, contact details will be obtained. The adolescent will be contacted by a RA, who will briefly explain the research. If the adolescent agrees to participate they will be sent a link via email and text message containing a participant information sheet, a consent/assent form, and the baseline adolescent battery. Recruitment is expected to be completed in Summer 2026.

## Methods: assignment of interventions

### Randomisation

#### Sequence generation

Eligible participants who provide written informed consent will be randomly assigned to one of two groups: the APT intervention group or the waitlist control group. The randomisation unit is the family of the consulting parent. The randomisation process will employ a 1:1 allocation ratio. Restricted stratified block randomisation is used with three blocking variables: consulting parent’s age, gender and education. These variables were significantly related to the intervention’s outcome in the previous pilot study.

#### Allocation concealment mechanism

Following baseline data collection, the RA will contact the collaborating statistician to randomly allocate the participant to APT or WL. RAs will be blind to group allocation.

#### Implementation

The collaborating statistician will have access to the random allocation sequence and randomly allocate participants to APT or WL. The RA will enroll participants into the study. The RA will not have access to the random allocation sequence. The random allocation list will be stored on a secure electronic database, compliant with data protection regulations to ensure confidentiality and integrity.

#### Blinding

An independent statistician will manage the randomisation process to ensure impartiality. Due to the nature of the waitlist control design, participants and therapists will not be blinded to allocation. However, to maintain the integrity of the study, RAs responsible for collecting outcome data will remain blind to group allocations. This blinding reduces the risk of bias during data collection and ensures the reliability of the results.

#### Data collection methods

All data is collected by the RA. RAs involved in data collection will receive standardised training on study protocols, ethical considerations and administration of measures. For both parents and adolescents in the APT treatment group, data are self-reports completed by the parent or adolescent and collected at four time points: T1 (pre-intervention) baseline, T2 (after six sessions, end-of-treatment), and follow-ups at both T3 (three) and T4 (six months) following the end of treatment. For WL participants data are self-reports completed by the parent or adolescent and collected at six time points. T1 (Following randomisation), T2 (six weeks later), T3 (three months – end of WL beginning of treatment), T4 (end- of-treatment) and follow-ups at both T5 (three) and T6 (six months) following the end of treatment. Participating parents will be offered the choice of completing the battery in a paper or online format.

Data collection instruments for parent measures include the primary outcome measure: SIPA [17], which has excellent internal consistency and content validity [18].

Regarding the secondary outcome parent measures: The SDQ is effective in detecting mental health problems [19]. The PHQ-9 demonstrates strong psychometric properties [20, 21], as does the GAD-7 [22, 23]. The APQ-SF shows good criterion validity in differentiating clinical and nonclinical groups [24–26]. The CBQ has good discriminant validity in distinguishing between distressed and non-distressed families [27]. The Parenting Scale has good psychometric properties for measuring parental discipline styles, with internal consistency and validity, including in clinical populations [28, 29]. The RFQ has demonstrated adequate reliability and validity [30]. The WAI-SF-C shows excellent psychometric properties, with Cronbach’s alphas ranging from 0.85 to 0.90 for subscales and 0.91 to 0.92 for total scores [31, 32]. The Goal Based Measure is sensitive to change and valid for tracking progress towards goals [33].

For the adolescent battery, the SDQ-YP, APQ-YP, and RFQ demonstrate comparable psychometric properties to their parent-report counterparts [19, 24–26, 30]. Adolescent batteries will be completed online, and adolescents will be compensated with a £10 e-voucher for each completed test battery.

Therapists will complete a battery at the end of treatment. The psychotherapist battery includes the WAI-SF-T [31, 32]. APT supervisors will complete an observer battery on a randomly selected session’s video for participants who have consented to video recorded sessions. The observer battery includes Adherence to the APT model. Data collection forms can be accessed at UCL data repository.

To promote completion of follow ups and prevent missing data, participants will be informed of the timeline of the research study, that questionnaires will be completed at the start of the study, 6 weeks later, at 3 months follow up, and at 6 month follow up. Participants will be informed that if they are allocated to the waiting group, questionnaires will also be completed at the end of treatment, and at 3 and 6-month follow ups. To promote completion, the RA will phone parents the week before follow-up questionnaires are due, reminding participants that this is an essential part of the research to be completed at certain time points, and ask participants to re-score goals, which are sent via email. The RA will contact the parent weekly until questionnaires and goals are returned. Adolescent participants are sent questionnaires the week before measures are due, the RA will contact the YP up to 3 times (weekly) if these are unreturned.

#### Data management

All data will be collected through online forms completed by participants to reduce errors associated with data entry. Open Door is committed to safeguarding the privacy of their users and the same privacy rules will be applied to participants of this study. All data will be kept for a maximum of seven years after the study has ended and will be stored in a GDPR secure data management service. All data will be collected and stored in accordance with the Data Protection Act 2018. If a participant withdraws from the study, they can request to have their data and contact details deleted or allow us to keep the data. In the case of keeping the data, the research team will use the minimum personally-identifiable data possible to safeguard participants' rights (i.e. delete contact details and keep anonymous questionnaire data). Video recordings are for the purposes of maintaining quality of treatment and providing supervision to therapists. Participants will be informed of how video recording will be used and consent for video recording will be sought before taking part in the study. Recordings will be kept on a securely stored, encrypted file and will only be used by participants' therapist and their supervisor during supervision and will not be shared with anyone else. After completion of therapy, all videos will be deleted. Participants can withdraw consent for video recording at any time, at which point all videos will be deleted.

### Statistical methods

The primary analysis will compare the effectiveness of APT to the waitlist control condition, focusing on the SIPA scores at post-intervention. The primary outcome variable will be analysed using between-groups mean differences, and effect sizes will be calculated for the end-of-treatment and three- and six-month follow-ups. Outcomes will be predicted by mixed-effects linear regressions with special interest in allocation group and baseline group differences (if any). Secondary outcome variables will be analysed using mixed-effects models (linear and nonlinear, logistic or Poisson as appropriate). Partner attendance, adolescent attendance, working alliance and reflective function will be assessed in their role as predictors and/or mediators of change. The unit of analysis will be the referred parent, even when they have attended APT with their partner. Maintenance of effects will be examined by comparing outcomes at the six-month follow-up to post-intervention scores. An intention-to-treat approach will be employed for all analyses, ensuring that participants are analysed in the groups to which they were originally assigned, regardless of adherence to the intervention protocol.

## Methods: monitoring

### Data monitoring committee

A data monitoring committee is not planned due to the low-risk nature of this intervention. No interim analyses are planned for this trial. Decisions to modify the trial will be made by the lead researcher, trial coordinator, trial sponsor, and REC.

### Trial monitoring

The study will be monitored by the CI (chief investigator), Dr. Alex Desatnik, and safety and efficacy will be reviewed by the CI and study co-applicants on a monthly basis. The research team will meet monthly in order to review study progress, and address any barriers or problems. Additional study oversight will be done by UCL Research Department of Clinical, Educational and Health Psychology. Ongoing data monitoring will be provided by an independent statistician. All research team members have completed training in data confidentiality either at Open Door, or another academic institution as part of the terms of their employment.

### Ethics 

#### Research ethics approval

Ethical approval for the RCT has been granted by the NHS ethics board Research Ethics Committee (REC) reference: 18/SC/0613, IRAS project ID: 254697.

#### Protocol amendments

Substantial protocol amendments will be submitted for approval to the REC and trial sponsor prior to implementation. Approved changes will be updated on the trial registry where applicable.

#### Consent or assent

The research team is aware that the study concerns vulnerable individuals, and potential risks and burdens have been carefully considered. The RA will obtain consent from participants. The RAs have received standardised training on study protocols and ethical considerations. Once the service user agrees to participate in the study, a phone call will be arranged with a RA to explain the study, obtain preliminary consent to be randomised, and complete preliminary consent procedures. A RA will then arrange an appointment to provide more information to the parent and sign informed consent forms. Participants will have the opportunity to ask questions and will be asked for consent for sessions to be filmed, which is optional. Adolescents who agree to participate will sign a consent/assent form. Adolescents will be informed that consent or assent can be withdrawn at any time, with no negative consequences to the adolescents or their parents. Proxy consent is not applicable for this study as all potential participants have decisional capacity to participate.

#### Confidentiality

Information collected in this study is sensitive, including records relating to participants' mental health and wellbeing and video-taped therapy sessions. Service users have no obligation to take part if they do not want their data to be used. Data collected during the study will be anonymised and will contain only a unique participant number as identification. A securely stored, encrypted file will contain the personal data associated to each participant, accessible only by the research team. Any paper-based information will be kept in locked storage at Open Door and will only be accessible by the research team and will be destroyed 6 months after the study has ended. No identifiable data will be transmitted to sponsors, co-investigators, and external parties.

#### Ancillary and post-trial care

If participants require further intervention following the completion of the study, they may have a discussion with their therapist, who can arrange continued provision of other intervention services in Open Door or refer them to other service providers if necessary.

## Discussion

This study aims to provide robust evidence on the effectiveness of a brief, individualised parenting intervention—APT—for parents of adolescents experiencing clinically significant levels of stress. By including a control group and a follow-up period, the trial addresses key limitations of the earlier pilot study [14]. If demonstrated to be effective, APT could serve as an accessible and engaging intervention, supporting parents and ultimately contributing to improved outcomes for adolescents and their families.

The key strength of this study lies in its pragmatic trial design, conducted in a real-world clinical setting, which enhances the ecological validity of the findings. The inclusion of both parent- and adolescent-reported outcomes adds depth and breadth to the evaluation, capturing multiple perspectives on the impact of the intervention.

However, this study has some limitations. The use of waitlist controls, while addressing ethical considerations, does not account for non-specific treatment effects, which could impact the interpretation of the findings. Future research could build on this design by comparing APT with an active control condition, enabling a more precise understanding of the specific mechanisms of action of the intervention.

Overall, this trial represents a significant step forward in the evidence base for parenting interventions targeting parents of adolescents. Given the well-established influence of parenting on adolescent outcomes, effective and accessible interventions in this domain have the potential for substantial public health benefits. Findings from this study will not only inform clinical practice but also contribute to policy decisions and broader implementation strategies, amplifying its impact on adolescent mental health and wellbeing.

## Supplementary Information

Supplementary Material 1.

## Data Availability

No datasets were generated or analysed during the current study.

## Abbreviations

APQ: Alabama Parenting Questionnaire
APQ-SF: Alabama Parenting Questionnaire-Short Form
APQ-YP: Alabama Parenting Questionnaire- Young Person Version
APT: Approach for Parenting Teenagers
CBQ: Conflict Behaviour Questionnaire
CCG: Clinical Commissioning Groups
CEO: Chief Executive Officer
GAD-7: Generalised Anxiety Disorder Screener
GDPR: General Data Protection Regulation
ICH GCP: International Council on Harmonisation of Technical Requirements for Registration of Pharmaceuticals for Human Use Good Clinical Practice
NHS: National Health Service
PHQ-9: Patient Health Questionnaire, 9-item depression module
RA: Research Assistant
RCT: Randomised Controlled Trial
REC: Research Ethics Committee
RFQ: Reflective Function Questionnaire
SDQ: Strengths and Difficulties Questionnaire
SDQ-YP: Strengths and Difficulties Questionnaire- Young Person Version
SIPA: Stress Index for Parents of Adolescents
UCL: University College London
WAI-SF-C: Working Alliance Inventory-Short Form-Client
WAI-SF-T: Working Alliance Inventory-Short-Form-Therapist
WL: Waitlist
