# Supplementary material for: Study protocol for a randomised evaluation of a brief psychological intervention for clinically stressed parents of adolescents: Approach for Parenting Teenagers (APT)
Source: BMC Psychol. 2026 Mar 21;14:637. doi: 10.1186/s40359-026-04048-w (PMC13130617; doi:10.1186/s40359-026-04048-w)
Supplement: Supplementary file 1 — Supplementary Material 1. [file 40359_2026_4048_MOESM1_ESM.docx]

Administrative Information

*Title and Structured summary*

**Scientific Title:** Study Protocol for a Phase I Superiority Randomised Trial Evaluating the "Approach for Parenting Teenagers (APT)" Brief Psychological Intervention Versus a Waiting List Control in Clinically Stressed Parents of Adolescents: Primary Outcome – Reduction in Parental Stress

**Public Title:** Study protocol for a randomised evaluation of a brief psychological intervention for clinically stressed parents of adolescents: Approach for Parenting Teenagers (APT)

**Trial Registration:** NCT03916172 (see Open Science section)

**Sponsor:** Open Door Young People’s Service

**Funding:** APT has received funding for this study (see Funding and Conflicts of Interest section)

**Contact (Public and Scientific Queries):** a.desiatnikov@ucl.ac.uk

**Study Location:** United Kingdom

**Study Type**: Interventional, parallel-group, randomised, superiority trial (Phase I)

**Intervention:** Approach to Parenting Teenagers (APT), an individual parenting programme for clinically stressed parents of adolescents

**Date of First Enrolment:** 18/09/2018

**Target Sample Size:** 80 participants, divided equally between the APT intervention and waitlist control group

**Primary Outcome Measure:** Stress Index for Parents of Adolescents (SIPA)

**Secondary Outcome Measures:** Mediators of change and adherence to APT

**Ethical Approval:** NHS ethics board Research Ethics Committee (REC) reference: 18/SC/0613, IRAS project ID: 254697

**Inclusion Criteria:** Parents of adolescents aged 11-18 years, meeting clinical levels of stress

**Exclusion Criteria:** Receiving treatment for psychotic illness; have previously received the APT intervention; parents of adolescents with severe developmental disorders or life-threatening conditions; parents whose adolescents are receiving treatment at Open Door.

**Data Sharing:** Participants can request that the study results are shared with them.

*Protocol version*
Version 1 (date: 11/11/25)

*Roles and Responsibilities*

**Contributors:**
Dr Alex Desatnik, Lead Researcher, Open Door Young People's Consultation Service

Catherine Jones, Clinical Research Assistant, Open Door Young People's Consultation Service

Professor Peter Fonagy, Academic Oversight, University College London

Julia Britton, Clinical Oversight, Open Door Young People's Consultation Service

Victoria Hayward, Clinical Oversight, Open Door Young People's Consultation Service

Ruth Glover, Clinical Oversight, Open Door Young People's Consultation Service

Lara Taylor, Clinical Research Assistant, Open Door Young People's Consultation Service

Dr Nicolas Lorenzini, Trial Coordinator, Open Door Young People's Consultation Service

**Trial Sponsor Contact:**

Ruth Glover (CEO)

Phone: 020 8348 5947

Email: ruth.glover@opendooronline.org

Open Science

*Trial Registration*

This trial is registered at the International Council on Harmonisation of Technical Requirements for Registration of Pharmaceuticals for Human Use Good Clinical Practice (ICH GCP).

Clinical Trial NCT03916172

Date of Registration: April 16, 2019

URL: <https://ichgcp.net/clinical-trials-registry/NCT03916172>

*Protocol and Statistical Analysis Plan*

The trial protocol and full statistical analysis is accessible at the ICH GCP (see Trial Registration).

*Data Sharing*

Upon completion all relevant study materials will be made accessible at UCL data repository.

*Funding and Conflicts of Interest*

APT has received two rounds of direct monetary support funding from the Big Lottery and Haringey CCG. Open Door Young People’s Service, the developer of the APT intervention, is funding the study. No monetary or financial benefits will be gained by Open Door from the results of the study and we do not expect their involvement to affect the results or interpretation of the findings.

*Dissemination Policy*

Study findings will be disseminated widely, including publication in peer-reviewed journals and presentations at academic and professional conferences. Results will be shared with participants, service users, and stakeholders through accessible reports and presentations. These efforts aim to inform both clinical practice and future research in parenting interventions for adolescents.
